# *In vitro* chlorhexidine release from alginate based microbeads for periodontal therapy

**DOI:** 10.1371/journal.pone.0185562

**Published:** 2017-10-03

**Authors:** Malte Scholz, Thomas Reske, Femke Böhmer, Anne Hornung, Niels Grabow, Hermann Lang

**Affiliations:** 1 Department of Operative Dentistry and Periodontology, Rostock University Medical Center, Rostock, Germany; 2 Institute for Biomedical Engineering, University of Rostock, Rostock, Germany; 3 Institute for General Practice, Rostock University Medical Center, Rostock, Germany; 4 Institute for Biostatistics and Informatics in Medicine and Ageing Research, Rostock University Medical Center, Rostock, Germany; Brandeis University, UNITED STATES

## Abstract

Periodontitis is one of the most common infectious diseases globally that, if untreated, leads to destruction of the tooth supporting tissues and finally results in tooth loss. Evidence shows that standard procedures as mechanical root cleaning could be supported by further treatment options such as locally applied substances. Due to gingival crevicular fluid flow, substances are commonly washed out off the periodontal pockets. The evaluation of administration techniques and the development of local drug releasing devices is thus an important aspect in periodontal research. This study describes the development and examination of a new alginate based, biodegradable and easily applicable drug delivery system for chlorhexidine (CHX). Different micro beads were produced and loaded with CHX and the release profiles were investigated by high performance liquid chromatography (HPLC). The *in vitro*-demonstrated release of CHX from alginate based beads shows comparable releasing characteristics as clinically approved systems. Yet many characteristics of this new delivery system show to be favourable for periodontal therapy. Easy application by injection, low production costs and multifunctional adaptions to patient related specifics may improve the usage in routine care.

## Introduction

Gingivitis and periodontitis are infectious diseases caused by bacteria. Untreated periodontitis lead to the destruction of the tooth supporting tissues and may result in tooth loss. Worldwide, periodontitis is one of the most common infectious diseases. In the United states of America 46% of all adults aged ≥ 30 years (representing 64.7 million people) suffer from periodontitis.[1] Severe forms, with periodontal pockets deeper than 6mm, differ from 10% to 15% worldwide.[2] In Germany almost every second (52%) young adult (aged 35–44 years) in Germany suffers from a periodontal disease.[3] Due to the demographic change and relations between periodontitis and other common diseases as diabetes and cardiovascular diseases, there will be an increasing therapy demand.[3] [4], [5], [6]

In the oral cavity more than 500 different bacterial species can be found[7] but not all of them belong to pathogenic strains. Especially gram-negative bacteria and a narrow diversity of bacterial strains in total are closely linked with progressive tissue destruction. After adhesion and formation of bacterial biofilms on the root surfaces, bacteria stimulate the host defence and a release of proteolytic enzymes is initiated[8] which results in the loss of tooth supporting tissues and periodontal pocket formation.

The goal of current therapies is the long-term reduction of the bacterial load and inflammation. Therefore, optimizing oral hygiene and mechanically cleaning of the root surfaces is the standard procedure. Additionally, different chemical and physical methods, as antibiotics, disinfectants, host response modulating molecules or for example laser activation, can support the therapeutic effect and increase regenerative potentials.[9] Further periodontal destruction may be treated by surgical intervention or may ultimately result in removal of the tooth. [10] For more than 45 years chlorhexidine (CHX) and its derivate (-digluconate and -diacetate) have been successfully used in periodontal therapy, due to a high potency, modest side effects and no bacterial resistance formation.[11] Chlorhexidine-digluconate is a widely examined and used antiseptic in dentistry. It shows effective disinfection in the oral cavity against different gram-positive and negative bacterial strains and fungus. The usage ranges from prophylactic pre- and postoperative disinfection to therapeutic usage in periodontal therapy. Different forms of applications are available for the professional usage with concentrations ranging from 0.1% to max. 20%.

Slow releasing devices, for a long-term therapy, have been developed and clinically established with different effects.[12][13][14] One of the most examined releasing device is a gelatine based CHX containing chip (Periochip) which is placed in deep pockets and releases CHX over seven days.[15] The size of the device limits the usage to severe forms of periodontitis. Moderate and light forms cannot be treated prophylactically due to small pocket dimensions. An injectable, liquid and spreadable material would thus support the use in further indications. Injectable materials are available containing e.g. doxycycline (Ligosan), metronidazole (Elyzol) and CHX (Chlosite). Slow releasing devices are supposed to consist of biodegradable materials that have a neglectable effect on the host response system. In general medicine different substances as poly(-lactid-co-glycolic acid) (PLGA), hyaluronic acid, gelatine, chitosan or alginate were used for a wide range of indications and drug releases.[16–18]

For the treatment of periodontal pockets different effects need to be considered. The gingival crevicular fluid flow (GCF) in the periodontal pocket serves as a defence mechanism, which is a serum exudate carrying host defence molecules and cells.[19] The flow increases with inflammation and reduces as side effect the concentration of locally administered drugs.[20] The dimensions of pockets on the other hand limit the application of non-injectable devices as mentioned above.

None of the existing application forms is currently able to overcome both of these limitations. Thus, the development of a biologic inert system that releases drugs over a predictable period in a sufficient amount with adherence to local tissues and easy application into any pocket formations seems of need. Therefore, our objective was to investigate the releasing profiles of CHX loaded microbeads by using a HPLC system. To create a controlled delivery system, different influences of the CHX release kinetic needed to be detected.

## Materials

### Alginate beads

The production of alginate based microspheres is widely examined and various procedures show reliable results in encapsulation of different materials.[18], [21–23] The simplest methods are described as dripping methods, where alginate sol is dripped into a saline solution (i.e. 2% CaCl_2_).[24] The gelation of the alginate bead starts in contact with multivalent ions in form of ionic cross-linking. The drop size hereby determines the resulting particle size. To produce small beads different procedures were preliminarily tested. Two of them seemed suitable to produce a sufficient amount of beads in an average size range from 10–400 μm.

Following the internal gelation procedure, described by Poncelet et al[25], alginate beads with diameters of 50 to 1000 μm were produced. The mean particle size is about 200 to 500 μm. Smaller beads from approx. 5–150 μm were produced by using an ultrasonic spray technique developed by the author.[26]

### Internal gelation process

Sodium alginate (Sigma-Aldrich Chemie GmbH, Germany) was dissolved for 24h in H_2_O to achieve solutions of 2% w/w.

**Fig 1:**
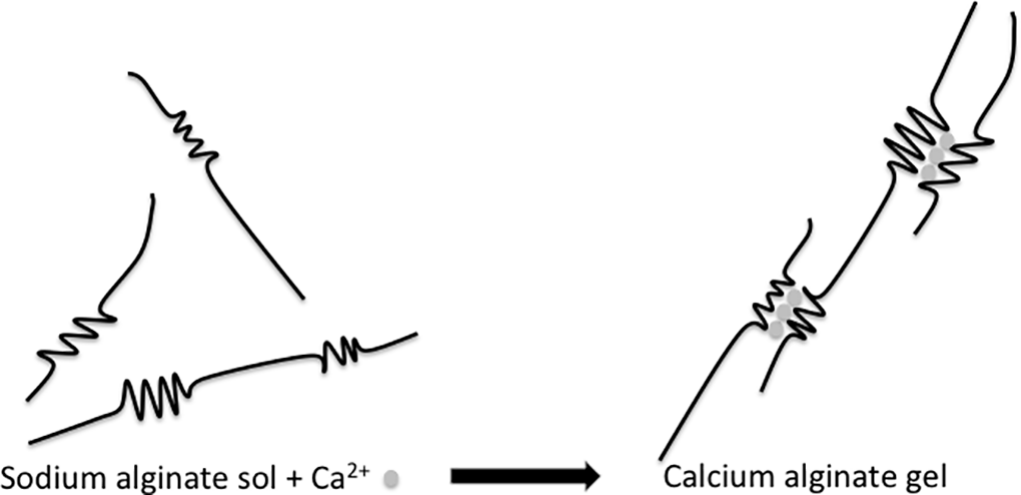
Alginate cross-linking. Ionic cross-linking of sodium alginate sol in contact with calcium ions.

**Fig 2:**
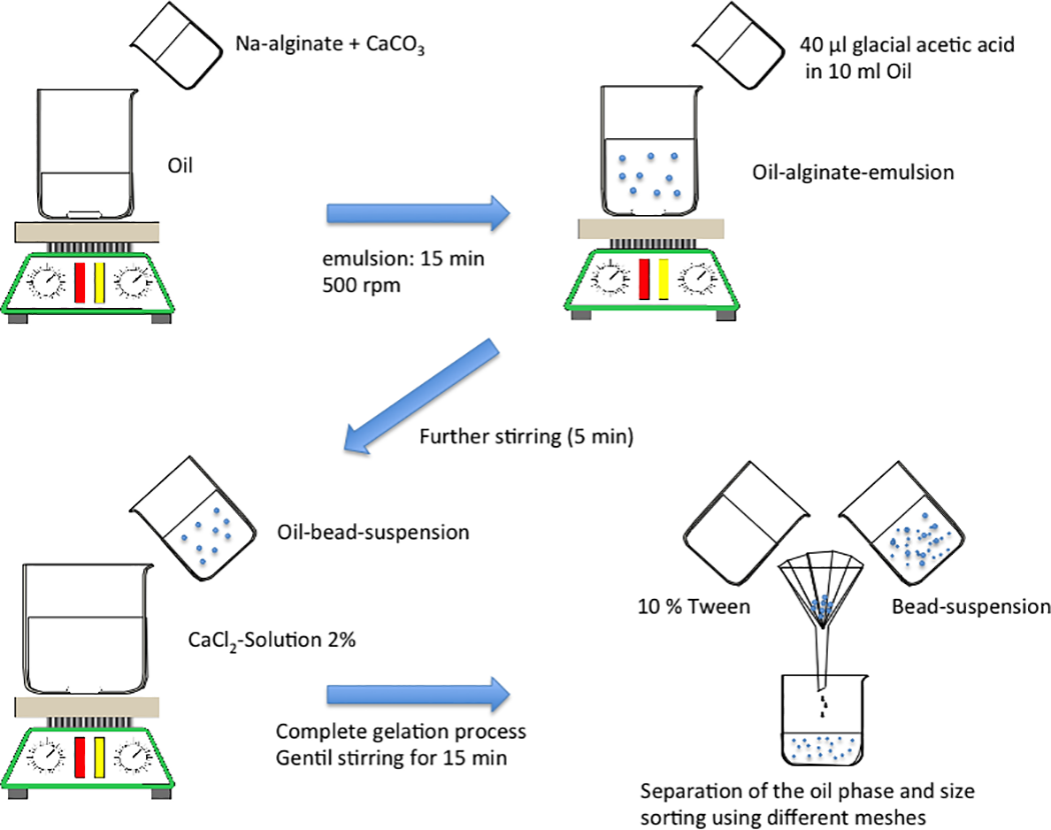
Internal gelation process. Schematic workflow of alginate beads production by internal gelation. Using emulsification and an internal initiated gelation to produce beads in a mean size of 200–500 μm.

**Fig 3:**
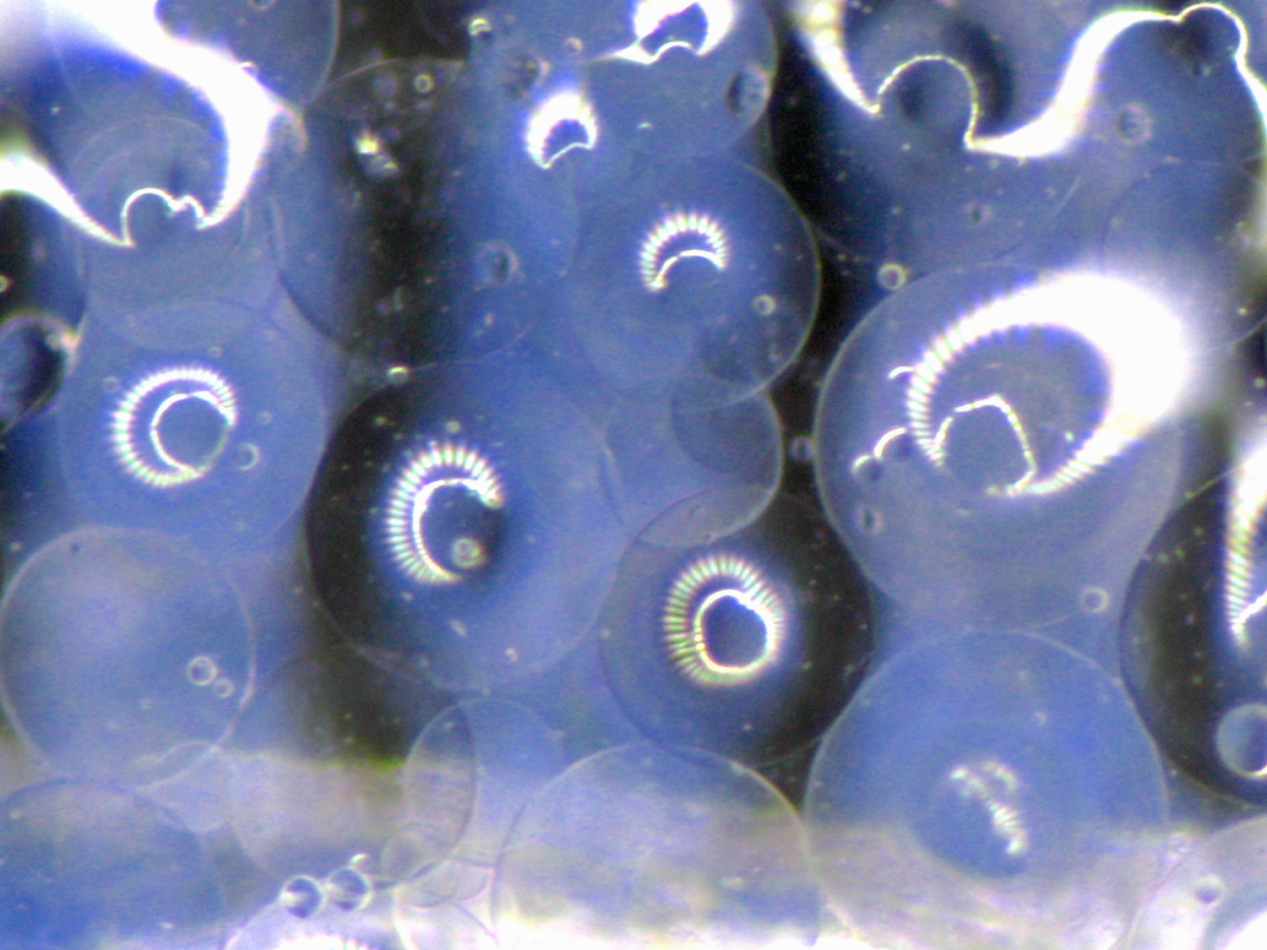
200–400 μm sized alginate beads. Light optical microscope picture of 200–400 μm sized alginate beads produced by internal gelation.

Hardly dissolvable calcium carbonate (Sigma-Aldrich Chemie GmbH, Germany) was suspended in H_2_O in a concentration of 500 mM. Furthermore a calcium chloride (Sigma-Aldrich Chemie GmbH, Germany) solution (50 mM) was prepared. Vegetable oil (Mazola, Kraft Foods, Germany), glacial acetic acid (Sigma-Aldrich Chemie GmbH, Germany) and Tween 20 (Polysorbate 20, Aldrich, 10%) were purchased and used at ambient temperature. 10 ml of alginate sol were mixed with 0.5 ml of calcium carbonate solution. The mixture was added to 50 ml vegetable oil and stirred for 15 min with a stirring speed of 500 rpm. During that time the alginate sol was emulsified in the oil-phase. Alginate bubbles were produced in different sizes. To initiate the internal gelation process, 40 μl of glacial acetic acid were added to 10 ml oil and given into the oil-alginate emulsion and stirred for 5 min. The glacial acid caused the dissolvation of calcium carbonate in the alginate and released Ca^2+^-ions, which initiated the exchange of sodium- and calcium-ions. (Fig 1) Finally, the suspension was added to 75 ml of calcium chloride solution and stirred for further 15 min to achieve a complete gelation. After separation of the oil phase, the alginate beads were washed using Tween 20 solution. The beads were sorted in size ranges from 100–200 μm and 200–400 μm by using stainless steel filters. (Figs 2 and 3)

### Ultrasonic spray technique

**Fig 4:**
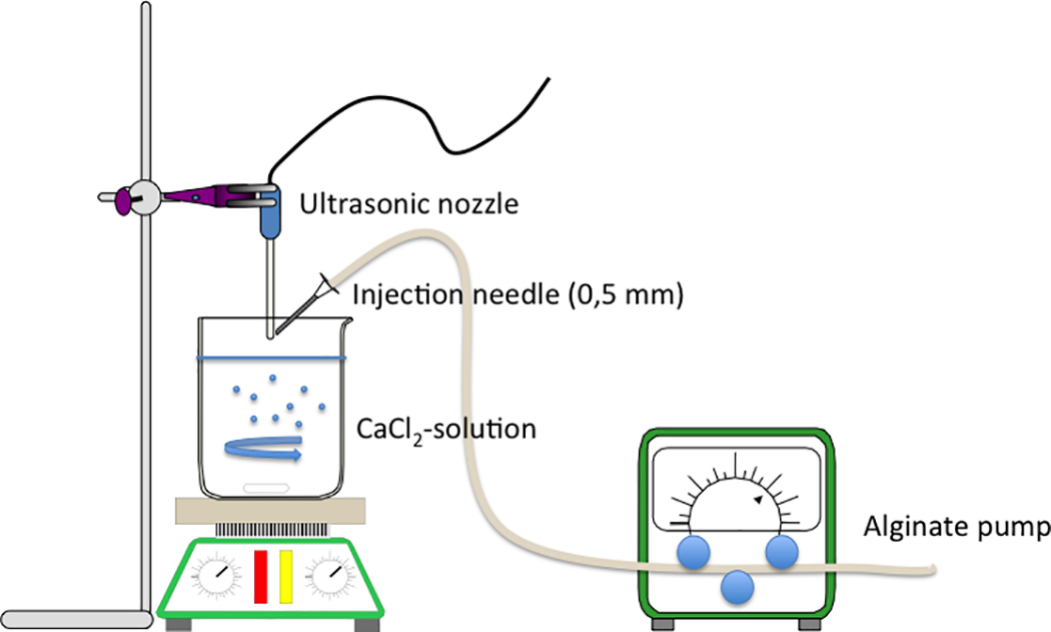
Ultrasonic spray technique. Schematic workflow of alginate beads production by ultrasonic activation.

**Fig 5:**
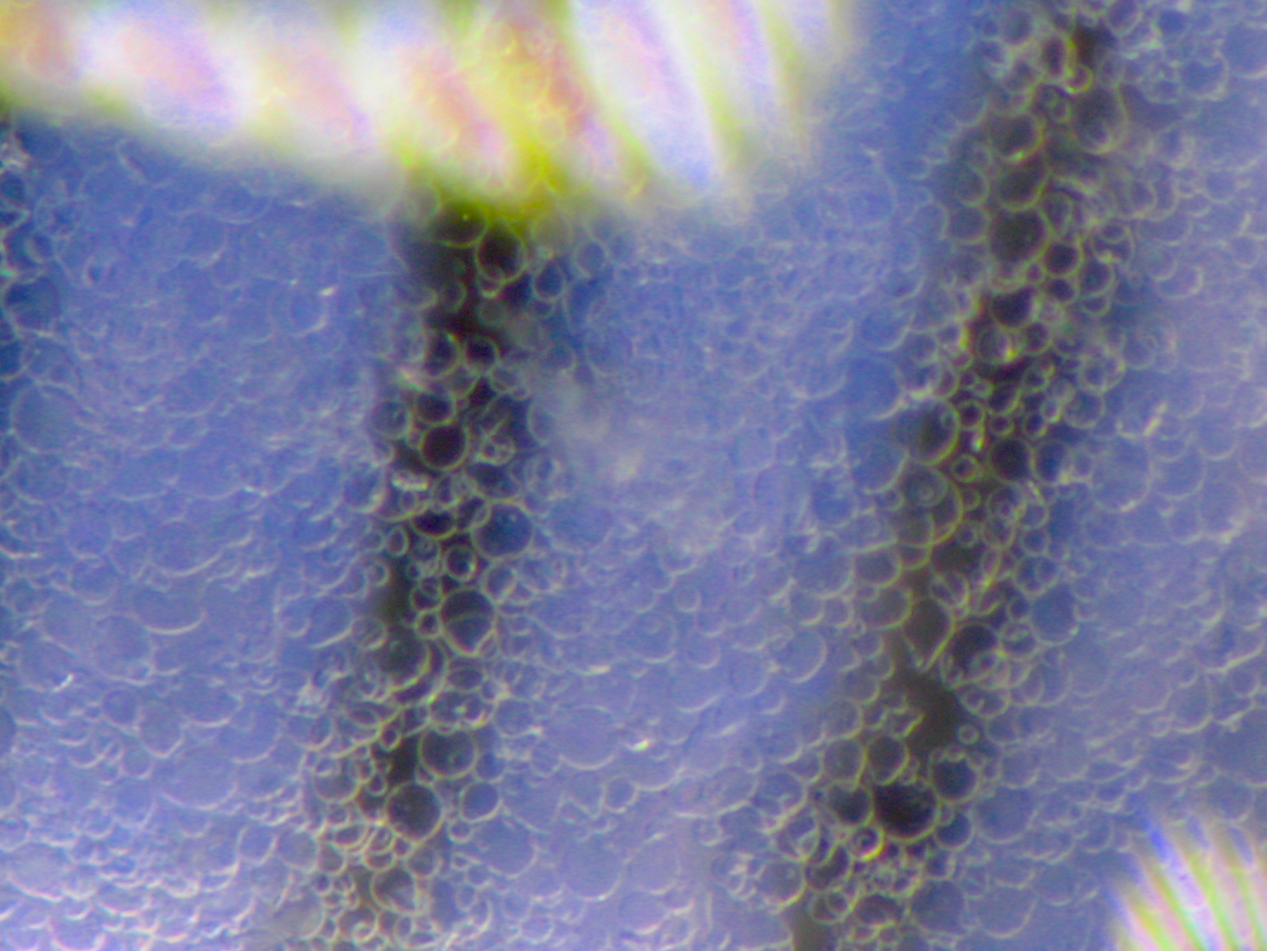
50 μm sized alginate beads. Light optical microscope picture of 50 μm sized alginate particles produced by ultrasonic activation.

Sodium alginate (Sigma-Aldrich Chemie GmbH, Germany) was dissolved for 24h in H_2_O to achieve a solution of 2% w/w. 200ml of 3% CaCl_2_-solution were prepared in a 500 ml beaker and stirred with a magnetic stirrer at a speed of 300 rpm. The ultrasonic activation was performed by using a dental scaler (EMS, Switzerland). The ultrasonic nozzle was placed at a distance of 3 cm to the surface. The alginate sol was pumped and transported to the nozzle´s tip with a needle of 0.5 mm diameter. Alginate sol was dispersed by the ultrasonic energy into spray drops and fell into the CaCl_2_-solution. Here, the gelation process was initiated externally by the exchange of sodium- and calcium-ions in the alginate. The produced particles were filtered and sorted by size using stainless steel filters at a size range of 20–70 μm to achieve a mean size of 50 μm. (Figs 4 and 5)

### Alginate blocks

Alginate blocks were produced by pouring 3% alginate sol into a square cast (1x1 cm) and covered with 6% CaCl_2_-solution. Complete gelation was reached after 24h and finally cut mechanically in preferred sizes.

### Poly(-lactic-co-glycolic acid) (PLGA) based particles

PLGA based particles produced in evaporation technique with a mean size of 50 μm were purchased from micromod GmbH, Warnemünde, Germany.

### Artificial saliva

The release of CHX was set to be determined in artificial saliva. Standard artificial saliva contains sorbic acid as preservative agent. In the UV spectra of sorbic acid and CHX a maximum appears in the same range, what is disadvantageous for a distinct detection of CHX release. Thus, a sorbic acid free saliva was prepared by the pharmacy of the medical department, Rostock University Medical Center, Germany (Table 1).

**Table 1:** Content of artificial saliva.

Reagents	Weight per 100g
Potassium chloride	0.12 g
Sodium chloride	0.085 g
Sodium hydrogen phosphate	0.25 g
Sorbitol solution 70%	4.3 g
Calcium chloride-0.15%-magnesium chloride-0.05%- solution	10 g
Carmellose-sodium 400 (Blanose 7MF)	0.5 g
Water	84.645 g

Artifical saliva free of sorbic acid was used for HPLC-measurements and prepared with reagents listed above.

### Chlorhexidine

Water based chlorhexidine-digluconate (20%) was purchased from Sigma-Aldrich Chemie GmbH, Germany and used in different concentrations by dilution for the following experiments.

### Reference materials

To obtain a reference and comparable material, two commercially available slow delivery devices for CHX were purchased. The first is called Periochip and was purchased from Dexcel-Pharma, Alzenau, Germany. It is a gelatine based biodegradable slow-releasing-device containing 33% (2.5 mg) CHX. This small chip (5 mm x 4 mm x 0.35 mm, 7 mg) is placed in periodontal pockets and releases CHX for over 7 days in clinical settings. [15] The second is a xanthan based gel, called Chlosite, containing 0.5% chlorhexidine- digluconate and 1.0% of–dihydrochlorite. Chlosite was purchased from Ghimas, Casaleccio di Reno, Italy. It is used for an adjunctive periodontal treatment and supposed to release chlorhexidine over a period of 3 weeks as per manufacturer’s specifications.

## Methods

### Drug incorporation

Due to the multivalent ionic structure of the CHX molecule it was impossible to incorporate it into the alginate solution without initiating a gelation process. The beads were hence produced as described above and in a second step stored in CHX solution to achieve a drug loading by diffusion. Different concentrations and loading times were examined.

### *In vitro* drug release—study design and performance

To investigate drug release a High Performance Liquid Chromatography System (HPLC) from Knauer, Berlin, Germany was used under the following conditions: column Eurospher 100 C18, 250 x 4 mm, mobile phase: acetonitrile/0.08M NaH_2_PO_4_ + 0.5% triethylamine, pH 3.0 (350/650 v/v), isocratic flow rate: 1 ml/min, temperature: 20°C, detection wavelength: UV 251 nm, injected sample volume: 20 μl. The calibration was performed in a concentration range from 0.2 to 20 mg/l. Calibration standards are listed in Table 2.

**Table 2:** Calibration standards of HPLC.

**Column:**	Eurospher 100 C18, grain size 5 μm, 250 x 4 mm ID
**Mobile phase:**	acetonitrile/0.08M NaH_2_PO_4_ + 0.5% triethylamine, pH 3.0 (350/650 v/v) isocratic;
**Temperature:**	20°C
**Flow rate:**	1.0 ml/min;
**Detection:**	UV, 251 nm;
**Sample volume:**	20 μl
**Retention time:**	ca. 5.2 min
**Dilution:**	1:1 Methanol
**Calibration:**	10 mg chlorhexidine diacetate diluted in 10 ml H_2_O
**Calibration standard:**	Prepared by dilution of artificial saliva
**Concentration range:**	0.2–20.0 mg/l.

Each probe was weighed into 10 mg samples and added to 600 ml of artificial saliva. Samples were stored at 37°C and shaken for 100 rpm. The time of assessment was chosen individually for the following experiments.

To determine the residual drug loading the particles were stored for one week at 37°C in methanol. Then the CHX concentration was measured again by means of HPLC.

The stability of CHX in artificial saliva was tested successfully at ambient temperature and 37°C over a period of 7 days.

### Initial screening

An initial screening over a period of 140 h was performed with different alginate based single observations (see Table 3) to determine the specifications of more detailed investigations.

**Table 3:** Samples for initial screening.

Sample	Material	Production procedure	Storage
1	Alginate block size: 0.5 x 0.5 cm (n = 1)	3% alginate, cured 6% CaCl_2_-solution	24 h in 20% CHX
2	Alginate beads 100–200 μm diameter (n = 1)	Emulsification; 2% alginate	24 h in 20% CHX
3	Alginate beads 200–400 μm diameter (n = 1)	Emulsification; 2% alginate	24 h in 20% CHX
4	Alginate beads 50 μm diameter (n = 1)	Ultrasonic; 2% alginate	24 h in 20% CHX

Different alginate based samples were loaded for 24h with CHX for a screening investigation

### Release determination

After the first screening period, further investigations were planned and conducted. Hereby, longer investigation periods (up to 630 h) and higher sample sizes (n = 5) were used.

As reference two available CHX releasing devices, the Periochip and Chlosite, were used and the release rates within considered time intervals were evaluated for 630 h in total. The different investigations are defined in Tables 4–7. Three time intervals were defined for release determination; initial-release: t_i_ = 0–17 h, mid-term release: t_m_ = 17–177 h and late release: t_l_ = 177–393 h.

**Table 4:** Sample definition of alginate and PLGA beads.

Sample	Material	Production procedure	Storage
1	PLGA-beads 50 μm diameter (n = 5)	Evaporation	24 h in 20% CHX
2	Alginate beads 2% 50 μm diameter (n = 5)	Ultrasonic	24 h in 20% CHX

To compare alternative biodegradable materials, same sized alginate and PLGA beads were used and loaded in same way.

**Table 5:** Influence of different CHX-concentrations.

Sample	Material	Production procedure	Storage
1	Alginate beads 2% 50 μm diameter (n = 5)	Ultrasonic	24 h in 10% CHX
2	Alginate beads 2% 50 μm diameter (n = 5)	Ultrasonic	24 h in 20% CHX

Two different CHX concentrations were chosen to investigate the influence of different concentrated loadings.

**Table 6:** Influence of different loading times.

Sample	Material	Production procedure	Storage
1	Alginate beads 2% 200–400 μm diameter (n = 5)	Emulsification	1 min in 20% CHX
2	Alginate beads 2% 200–400 μm diameter (n = 5)	Emulsification	30 min in 20% CHX
3	Alginate beads 2% 200–400 μm diameter (n = 5)	Emulsification	6 h in 10% CHX
4	Alginate beads 2% 200–400 μm diameter (n = 5)	Emulsification	24 h in 20% CHX

Loading times from 1 min to 24h were chosen to show different release profiles.

**Table 7:** Combinations of different particle sizes.

Sample	Material	Production procedure	Storage
1	Alginate beads 2% 50/50% w/w (n = 5)	Ultrasonic: 50 μm diameter Emulsification: 200–400 μm diameter	24 h in 20% CHX
2	Alginate beads 2% 30/70% w/w (n = 5)	Ultrasonic: 50 μm diameter Emulsification: 200–400 μm diameter	24 h in 20% CHX

Combinations of different bead sizes were defined finally to investigate the effect of combining different release properties.

### Statistical analysis

All data were processed using IBM SPSS Advanced Statistics 23.0. All values are expressed as mean ± standard deviation (SD) for the indicated number of samples (n). To compare different groups of samples concerning release rates, nonparametric analysis of variance was performed employing the Kruskal-Wallis test (for >2 unpaired samples, otherwise the Mann-Whitney U-test, two-sided). If appropriate, subgroups were tested subsequently pairwise using the U-test. P-values < .05 (Bonferroni-adjusted for multiple testing) were considered to be statistically significant.

## Results

### Different alginate samples

**Fig 6:**
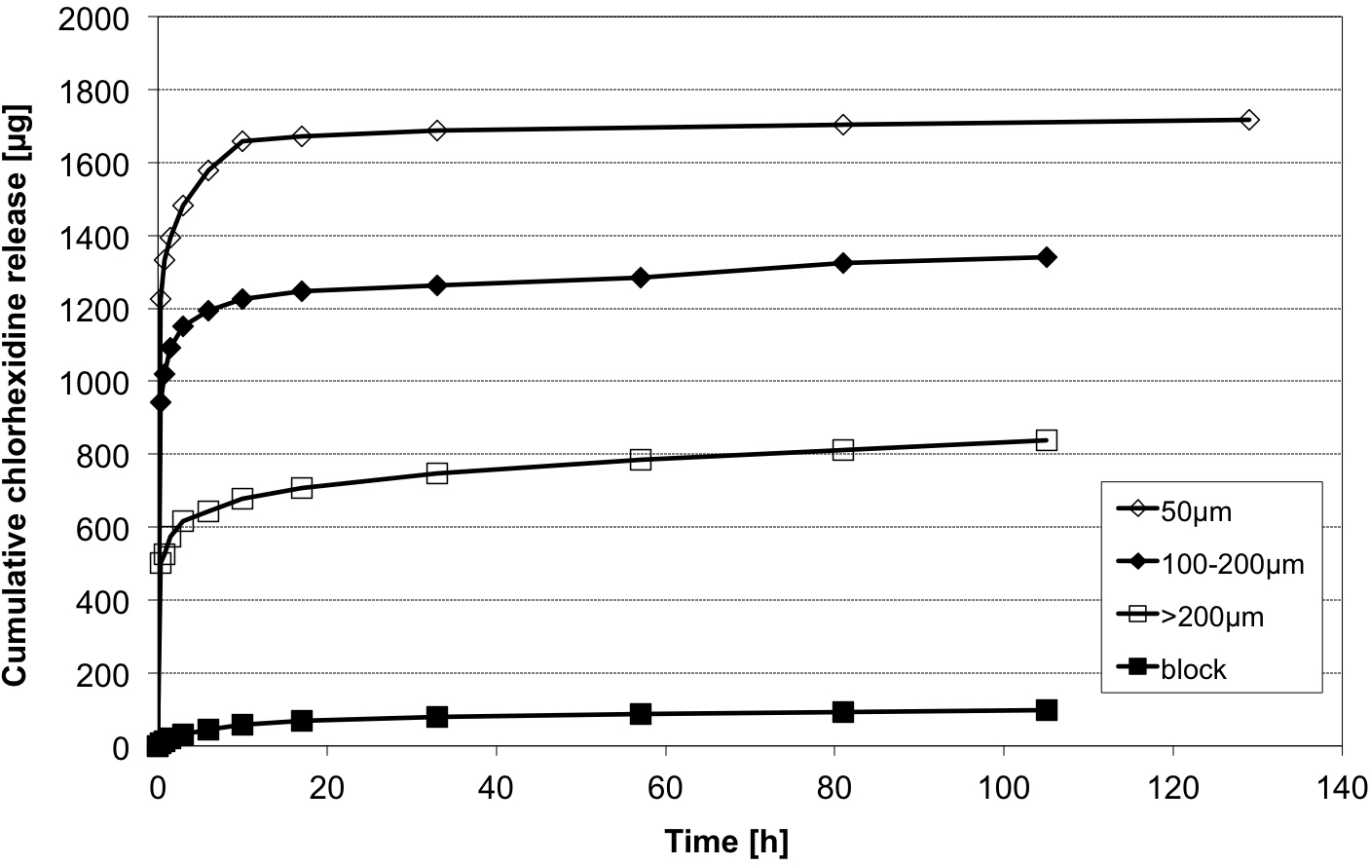
CHX release profiles of different sized alginate beads. Cumulative CHX release of initial screening measurements (HPLC) over 120 h (n = 1 samples each). CHX release was determined from different sized alginate based particles.

The initial screening showed different release profiles for different sizes of alginate probes, as shown in Fig 6. Hereby the alginate block released less CHX than all other particles. Respecting the largest surface with smallest diameter, the 50 μm particles released CHX faster than all other particles. After an initial burst release of the main amount of CHX within the first two days all particles showed slow release for the following two days. Particles sized larger than 200 μm showed the highest delayed release (t = 20 h to 105 h) with 130 μg compared to 96 μg in 100–200 μm group and 45 μg for 50 μm sized particles. That can be explained with a higher amount of CHX molecules binding to the inner alginate base in the larger beads. The initial release and total amounts of CHX were higher the smaller the particles are. To obtain high levels of CHX and higher long-term releases, we conducted to investigate beads sized 50 μm and 200–400 μm.

### Alginate vs. PLGA

**Fig 7:**
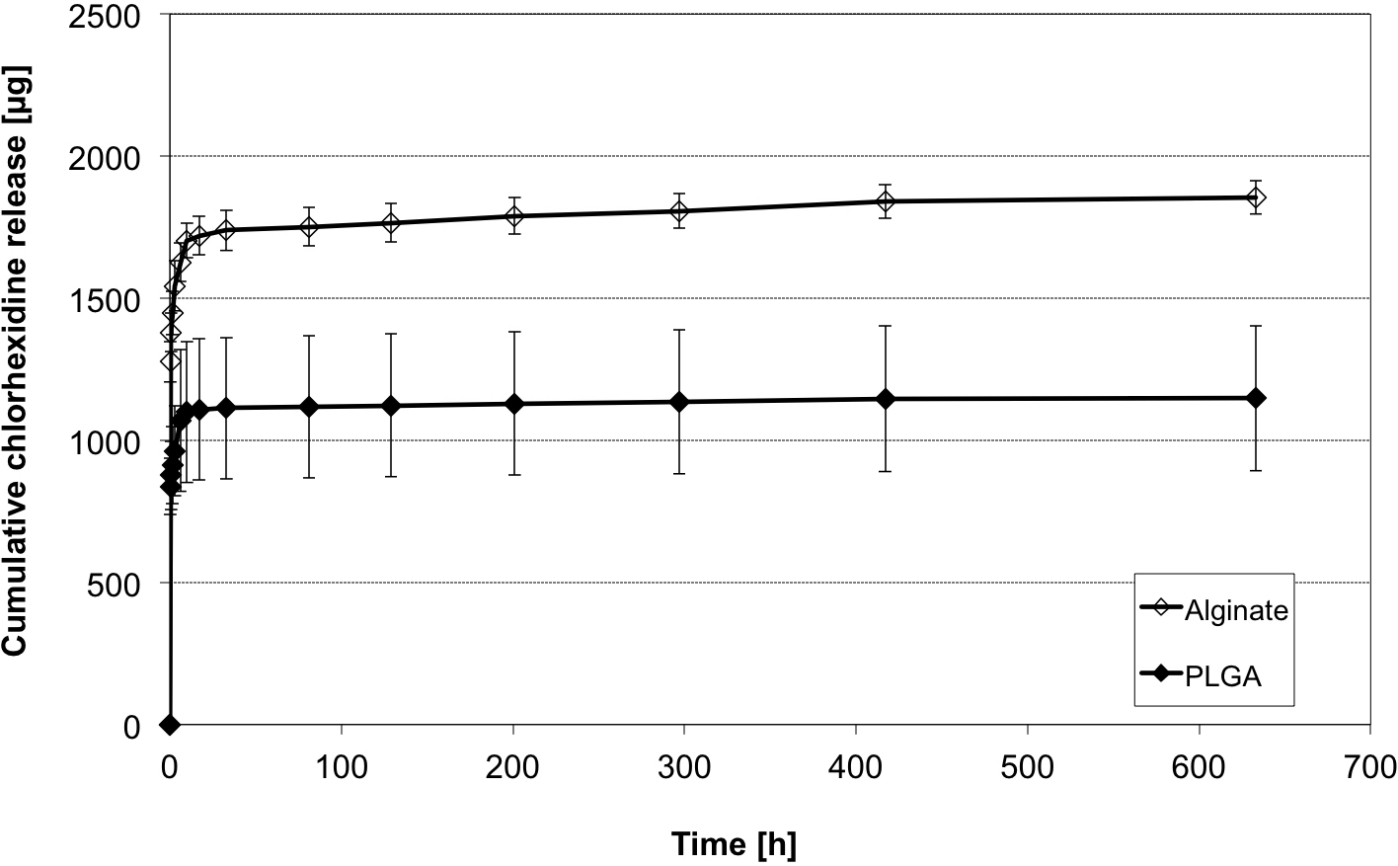
CHX release profiles of different biodegradable materials. Cumulative CHX release of 50 μm sized PLGA and 50 μm sized alginate particles over 630 h (mean ± SD, n = 5 samples each).

PLGA beads are commercially available and show predictable degradation profiles in the oral cavity. As a possible alternative for alginate PLGA was chosen, but did not show superior release characteristics (Fig 7). Both groups showed high initial release rates decreasing over the time. The alginate-particles released in both initial (t_i_) and mid-term (t_m_) release periods significant more CHX than the corresponding PLGA beads (p = .48, p < .001); in total 38.1% on average. There is only a very low release in long-term measurements between 10 and 630 h in the PLGA group (48.4 ± 7.1 μg), but the alginate showed a higher release of 151.6 ± 3.9 μg, which is about 8% of total released CHX. Due to a higher preparation complexity of PLGA-beads and the advantages of the alginate based particles the following investigations were conducted only with alginate based beads.

### Influence of different CHX-concentrations

**Fig 8:**
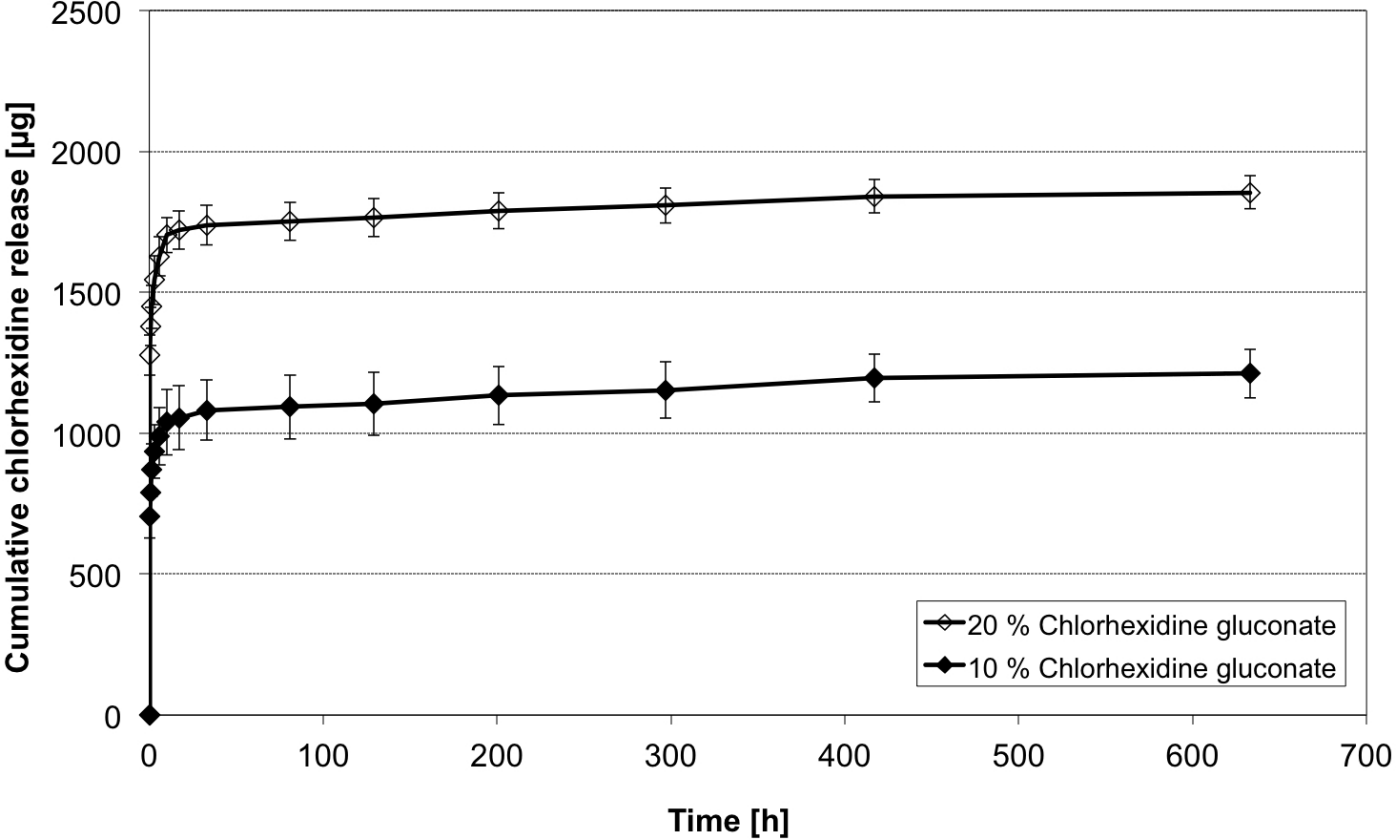
CHX release profiles of different CHX concentrations. Cumulative CHX release of over 630 h (n = 5 samples each). Loading 50 μm sized alginate based particles for 24h in 10% and 20% concentrated CHX. (mean ± SD, n = 5 samples each).

**Fig 9:**
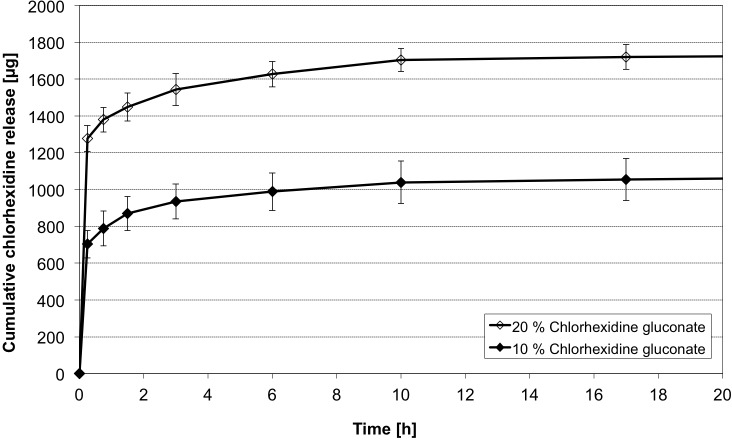
Initial CHX release profiles of different CHX concentrations. Cumulative CHX release of initial screening measurements (HPLC) over 20 h. Detailed view on the initial releasing profile of different loaded (10% and 20% CHX) particles. (mean ± SD, n = 5 samples each). (Compare Fig 8).

Dissolved CHX is only available in up to 20% aqueous solutions. Aiming on a high loading of CHX, only two different concentrations (20% and 10%) were investigated (Figs 8 and 9). Both groups showed almost identic release characteristics. They differed in the initial measured CHX concentrations. The group of higher concentrated CHX (20%) showed 1.8 times higher concentrations in the first measurement (t = 0.25 h). The factor was lowered during the time to finally 1.53 (t = 630 h). After the initial phase (t _i_) 134.7 ± 15.1 μg CHX were released on average in the CHX (20%) group, which is comparable to 156.9 ± 41.1 μg in the CHX (10%) group. The main difference is the early released amount of CHX, which is easily explained due to the higher concentrated CHX adhering to the surface of the micro beads. The following release follows similar kinetics and can be explained with the release of CHX-molecules binding to the alginate base.

### Influence of different loading times

**Fig 10:**
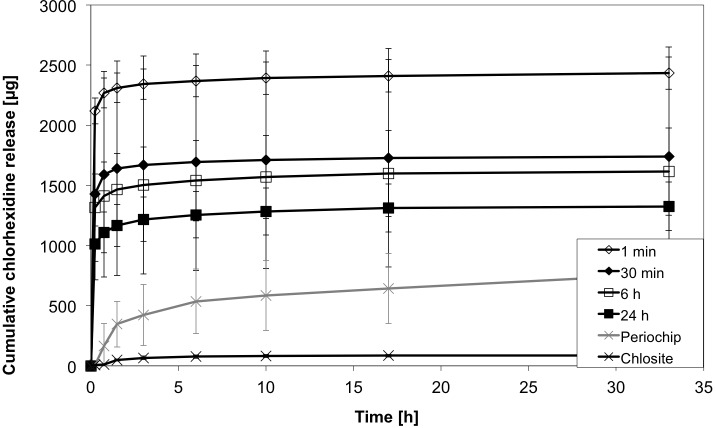
Initial CHX release profiles of different loading times. Cumulative CHX release over 35 h from 200–400 μm sized alginate beads loaded for 1 min, 30 min, 6 h and 24 h and compared with Periochip and Chlosite (mean ± SD, n = 5 samples each).

**Fig 11:**
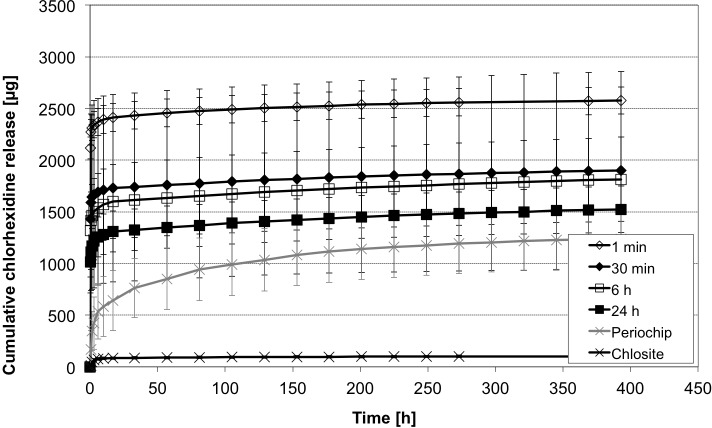
CHX release profiles of different loading times. Cumulative CHX release over 400 h from 200–400 μm sized alginate beads loaded for 1 min, 30 min, 6 h and 24 h and compared with Periochip and Chlosite (mean ± SD, n = 5 samples each).

The comparison of CHX release after different loading times (1min, 30min, 6h, 24h) of particles sized 200–400 μm showed an effective drug loading just after one minute of loading time (Figs 10 and 11). The highest initial release was found in the group of the shortest loading time (1min) with 2118.5 ± 106.4 μg, compared to 1014.9 ±146.0 μg in the 24h-group. These findings may be caused by more adhesion to the surface instead of loading and diffusion into the alginate base. For all considered release intervals (t_i_, t_m_ and t_l_) likewise, no differences in the release rates could be detected between the loading time groups (.138<p<1.0), reflecting a release behaviour independently of loading time for the alginate system. In contrast, the Chlosite system differs from all groups (p < .001, each interval), while the Periochip group differs from all groups merely in t_m_(p < .001) and from short-time loading (1min, 30min) in t_l_ (p = .025, p = .002).

### Evaluation of reference materials

**Fig 12:**
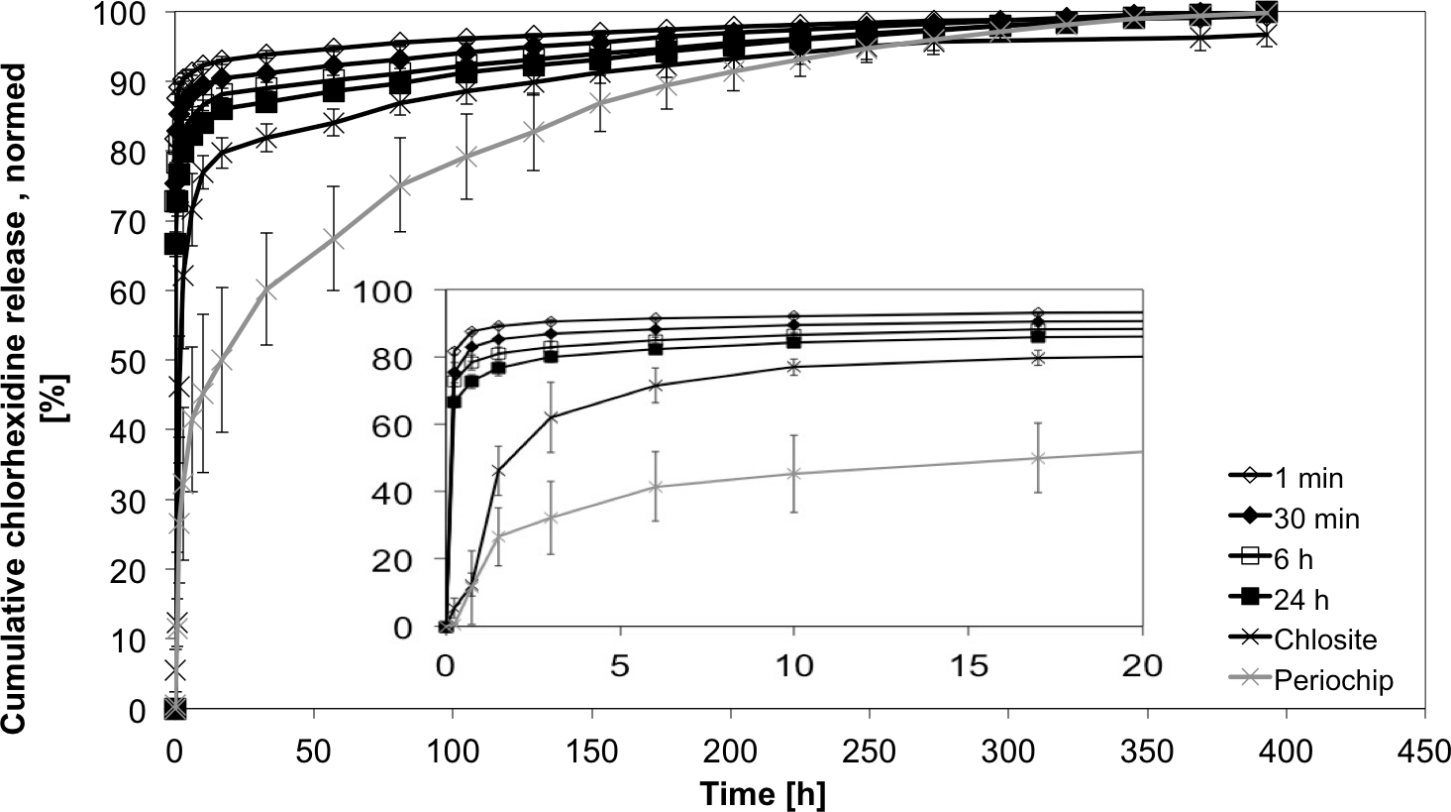
Normed CHX release profiles of different loading times. Normed cumulative CHX release over 400 h and 20 h from 200–400 μm sized alginate beads loaded for 1 min, 30 min, 6 h and 24 h and compared with Periochip and Chlosite (n = 5 samples each).(mean ± SD, n = 5 samples each).

In relation with the gelatine based Periochip and the xanthan based gel, Chlosite, the alginate beads showed a higher initial releasing profile (Fig 10). The total amount of released CHX at t = 393 h differed between considered groups, i.e. 1236.9 ± 280.4 μg for the gelatine chip vs. 102.2 ± 12.1 μg for the xanthan gel. But statistically significant could only be detected the difference of total release between the alginate (with 1min loading time) and Chlosite groups (p = .001). Especially the gelatine based chip showed a steady releasing profile over more than 300 h. Initially there was no release at t = 0.25 detectable. In the following period (t_i_) the release rose to almost 50% (641.7 ± 290.7 μg) of incorporated CHX in 17 h and finally up to 1236.9 ± 280.4 μg. In the alginate group the 50% range was reached in the first measurements, due to surface adhered CHX. The xanthan based gel showed a very low release of CHX at all (102.2 ± 12.1 μg). It reached the 50% range in the first 2 h and released a very low concentration in the later periods (Fig 12). In the periods t_m_ and t_l_ (from 17h to 393 h) only 16.5 ± 5.1 μg CHX were released, compared to 213.6 ± 46.4 μg from alginate beads loaded with CHX for 24h and 595.2 ± 123.1 μg in the gelatine chip-group.

### Combinations of different particle sizes

**Fig 13:**
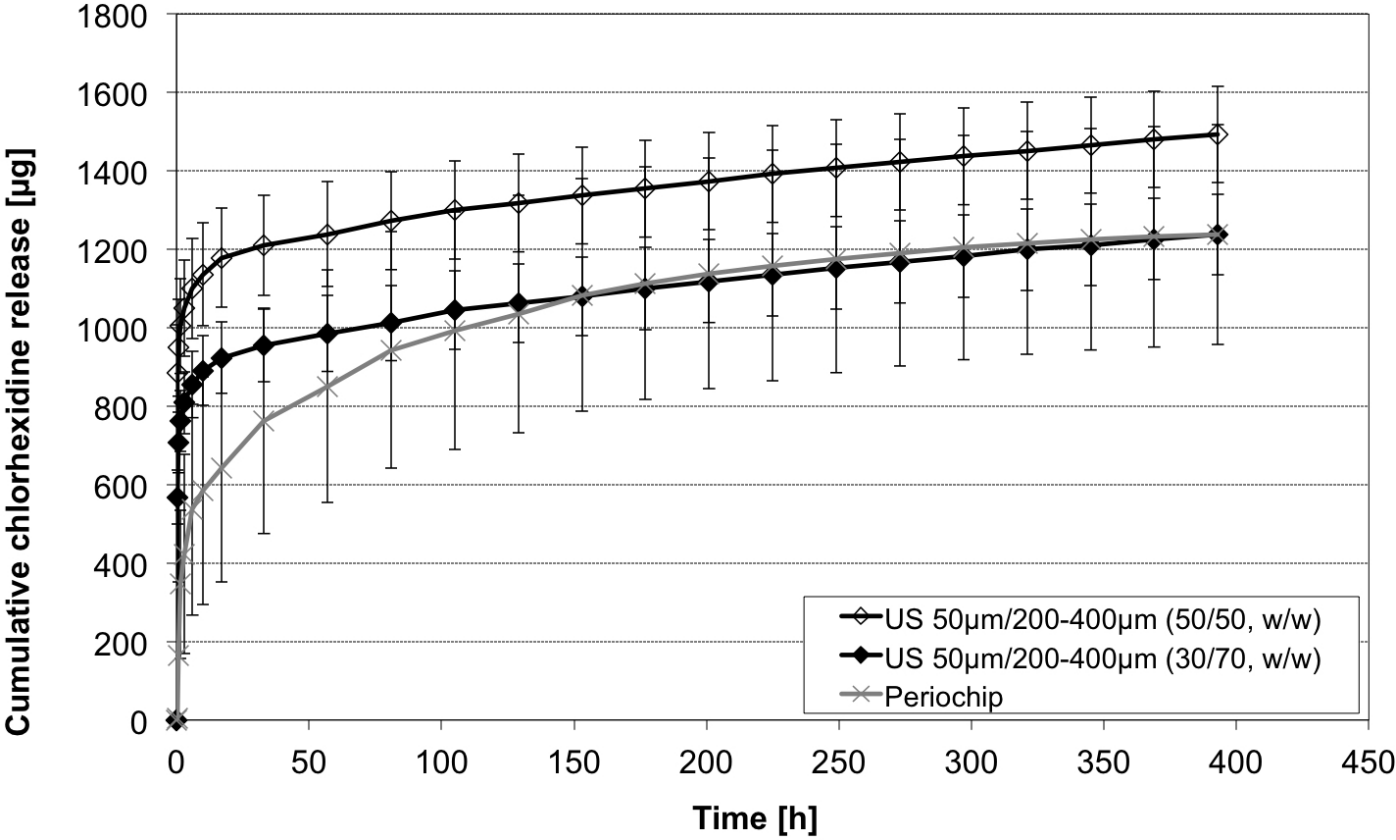
CHX release profiles of bead combinations. Cumulative CHX release over 400 h from alginate bead combinations, sized 50 μm and 200–400 μm in ratio 30/70 w/w and 50/50 w/w compared with Periochip (mean ± SD, n = 5 samples each).

**Fig 14:**
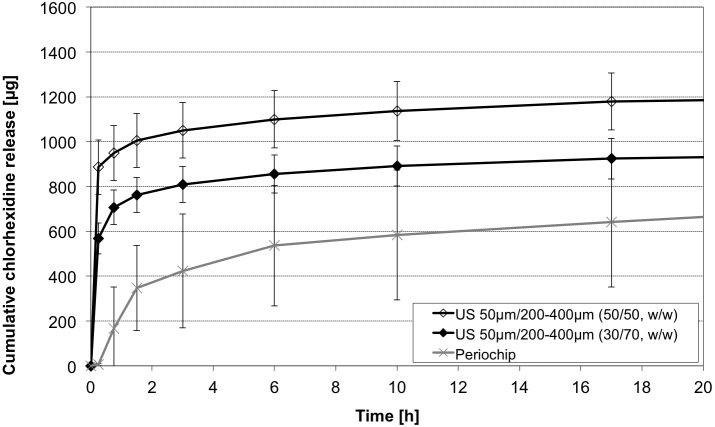
Initial CHX release profiles of bead combinations. Cumulative CHX release over 20 h from alginate bead combinations, sized 50 μm and 200–400 μm in ratio 30/70 w/w and 50/50 w/w compared with Periochip (mean ± SD, n = 5 samples each).

To obtain a release profile combining a burst release phase and a following long-term release of two different sizes, 50 μm and 200 μm– 400 μm, respectively, were mixed. Here lower standard deviations occurred compared with the gelatine chip, where differences up to 300 μg were detected (Fig 13). Over all an adaption of the long-term release of the alginate to the gelatine chip could be realised. In fact, the combination with higher amount of 50 μm-sized particles showed a slightly higher initial burst (t = 0–1.5h) compared to the “30/70 w/w” composition. But, during the early release. period(t_i_) all groups can be considered rather comparable concerning release (p = .967, Fig 13). After 393 h the total amount of CHX was for (50/50 w/w) 1492.1 ± 122.3 μg, (30/70 w/w) 1237.4 ± 102.7 μg and for the gelatine chip 1236.9 ± 280.4 μg (Fig 14). Hereby more than 50% of all CHX was released after an initial burst in the alginate compositions. The chip released more than 50% after 17 h observation time. Up to t = 150 h (30/70 w/w), (50/50 w/w) and the gelatine chip released no different amounts of CHX (1079.8 ± 100.7 μg, 1336.9 ± 122.9 μg and 1083.3 ± 296.4 μg, p = .065). From this time the curves followed an almost similar profile and a release of 157.6 ± 23.5 μg (30/70 w/w), 155.2 ± 14.7 μg (50/50 w/w) and 153.5 ± 35.8 μg (gelatine chip) was detected. The gelatine chip showed the most potent release during the mid term release phase (t_m_) (with 472.2 ± 97.4 μg), compared to both alginate combinations (p < .001 each, Fig 13) which is almost double as high as both other compositions.

## Discussion

This investigation shows alginate microbeads to be a successful delivery system for CHX. Even short loading times (1min) released effective concentrations, longer loading times prolonged a late release from the alginate base. For a sufficient disinfection of the periodontal pocket a high initial CHX quantity seems beneficial [27], which can be easily achieved with smaller particles. Larger particles released CHX more slowly in higher doses and thus obtained disinfection for longer periods. Combining different sized particles, 50 μm and 200–400 μm, respectively, modified the release profiles. By using these modalities the CHX release behaviour can be controlled and adjusted.

A major challenge in periodontal therapy that could be coped with our system is the effect of drug elution by gingival crevicular fluid flow, which reduces the concentration of locally administered substances. GCF differs disease related from healthy (3–8 μl/h) to infected sites (137 μl/h).[20] These effects show the necessity of a steady release in administrating medicaments in periodontal therapy. It seems advantageous to have a high initial burst that reduces the bacterial caused inflammation immediately, which can be achieved by our system. Using small particles and short loading periods obtain high initial released CHX (see Figs 13 and 14 and Figs 10 and 11). Reduced inflammation will result in reduced GCF flow and lower doses of CHX will be necessary to maintain disinfection.[28] In our *in vitro* investigation it is not shown how far GFC could transport whole micro particles out of the periodontal pocket. For further local stability the particles could be coated with or embedded into further biodegradable matrices to maintain adhesion between the particles themselves or the local tissues. This could result as well in further prolonged release characteristics, as shown by Matricardi et al.[29]

Two different biomaterials were primarily investigated to obtain a suitable micro bead matrix for CHX delivery. Alginate is a widely used and examined biomaterial with several indications in medicine as drug delivery, protein delivery, wound dressings, tissue regeneration and cell delivery.[18] Silva et. al showed suitable biochemical and biophysical properties, as degradation and biocompatibility, for the usage of alginate as drug delivery device in dentistry in degradation studies.[30] PLGA shows comparable characteristics in drug delivery and biocompatibility.[31] Releasing up to 40% less CHX we considered alginate as more suitable for further investigations.

CHX delivery is not only applied in dentistry—overall CHX is used to reduce bacterial colonisation of different tissues and surfaces in medicine. Especially in combination with alginate, wound-healing devices or vaginal applications have been investigated.[32], [33] Still, there is only little data for CHX release from alginate—no data about CHX release from alginate beads could be found in literature. Yet, Abruzzo et. al for example successfully developed chitosan and alginate complexes for vaginal CHX delivery.[34] In dentistry, multilayer membranes were investigated by Silva et al. as intraoral delivery devices for CHX.[30] Garner et al described the use of hexametaphosphate nanoparticles for the release of CHX in oral cavity.[35] Soriano-Souza et al used CHX loaded hydroxyapatite microsphere to proof osteo-conductive properties.[36] Here different effects were tried to combine: disinfection and regenerative potency. In wound healing Jiang et. al showed regenerative potency in combining the release of platelet-growth factor and CHX in PLGA microspheres.[37] These dual application forms could be realized by using alginate as carrier as well. All of these investigations support the promising translation of local CHX release to periodontal therapy.

Looking at clinical use, we compared our alginate system to Periochip. This gelatine based chip is a clinically approved device for CHX delivery in periodontal pockets as shown for example by Soskolone et al and Kalsi et al.[15], [38] Here, the releasing profiles of the gelatine chip and the combinations of alginate beads differed in the early and mid term release phase and were almost identical in later periods. This shows further the possibility to control and modify the CHX release profile from alginate beads. Higher initial CHX rates seem advantageous for bacterial elimination in the periodontal pocket [27]. The easier application form of the alginate bead system, compared to the bigger sized gelatine chip, can enhance the usage in larger groups of patients: the gelatine chip is supposed to be used in severe periodontal pocket formation deeper than 5 mm.[39] An injectable system could be used before periodontal destruction occurs by prophylactic application in minor pockets. Comparing the benefits of CHX releasing products in clinical studies these advantages seem promising for the implementation of the evaluated system into clinical settings. [40], [41]

Another strength of the alginate system may lie in the fact that *in vivo* it can be necessary to maintain controlled releasing grades of medicaments. In acute phase inflammations for example, obtaining high initial concentrations lasting for few days to reduce symptoms is favourable. For the prevention of bacterial colonization and supportive disinfection after mechanical therapy on the other hand, longer lasting lower doses of released CHX are preferable.[27] Therefore a patient related adaption of a modular releasing system would be of great use. This could be realized even chairside with the investigated alginate system. The releasing characteristics of the alginate based micro beads can be adapted as seen above by choosing different particle sizes, loading times, CHX concentrations, and finally particle combinations. The loading of 200–400 μm sized particles for only one minute would obtain effective disinfection characteristics for the treatment of acute periodontal inflammation (minimal inhibition concentration (MIC) for *porphyromonas gingivalis* 62 μg/ml).[42] Prolonged loadings and combinations of different particles seem more suitable for preventive indications like a reduction of bacterial recontamination of the periodontal pocket. After 150 h the released amounts of CHX in all groups were still higher than the MIC of most periodontal pathogenic strains.

The releasing profiles of alginate beads in our investigation can be easily adapted chairside individually for a patient and adapted to the inflammation status by using different sized particles, loading times, CHX concentrations and particle combinations. The implementation into clinical settings seems promising due to advantages as flexibility in handling, low production cost and adaption. Future aspects of our system may be combining a primary disinfection, followed by further inflammation control, with released medicaments[43] or probiotic bacteria[44] and the release of growth factors[45], stem cells[46] or proteins[47], to enhance regeneration in periodontal therapy.

## Conclusion

The release of CHX demonstrated *in vitro* from alginate based particles shows comparable release characteristics as clinical approved systems. The usage of alginate as a carrier for drug delivery is promising and advantages as being injectable, low production costs and a chairside patient related adaption make this system favourable for periodontal therapy and further improvement of clinical routine.

## Abbreviations

CHX: chlorhexidine
MIC: minimal inhibitory concentration
HPLC: high performance liquid chromatography
PLGA: poly(-lactic-co-glycolic acid)
GCF: gingival crevicular fluid flow
